# Recent application developments in genomic research using the AdvanCE™ FS capillary electrophoresis platform

**DOI:** 10.1186/1753-6561-5-S7-P50

**Published:** 2011-09-13

**Authors:** Julio Petersen, Dylan Baker, Deepak Dibya, Pierre Varineau, Jeremy Kenseth

**Affiliations:** 1 Advanced Analytical Technologies, INC

## 

Central to genomic research is the analysis of nucleic acids, which requires versatile, sensitive and high throughput technologies that can deliver accurate results in a short time. Reported herein are recent developments for sizing and/or quantifying nucleic acids (dsDNA and RNA) on a parallel capillary electrophoresis platform with LED-based fluorescence detection (AdvanCE™ FS system). This flexible multi-capillary platform has been employed for applications such as SSR, gDNA analysis, RNA analysis, quantification of next generation sequencing (NGS) libraries, and the separation and sizing of large cDNA from *Populus spp*,This poster will discuss recent validation of the platform for a subset of these applications in detail. The AdvanCE™ FS system successfully scored all SSR samples with resolution as low as 2 bp. Assessment of the quality/concentration of gDNA is also demonstrated with a new separation matrix, for separation of large DNA fragments. Separation and sizing of large cDNA was also demonstrated with good resolution and accuracy. A method for rapid and sensitive detection of total RNA concentration and quality will be discussed, as well as the sizing and quantification of NGS libraries.The AdvanCE™ FS platform offers rapid separation and ample resolution with excellent sensitivity and dynamic range, to benefit a variety of applications in genomic research.

